# Interfacility helicopter transfers for critically ill patients: always the right choice?

**DOI:** 10.1186/s13054-020-02846-1

**Published:** 2020-04-16

**Authors:** Hasan Hawilo, Ravi Taneja

**Affiliations:** Western University, London, Canada

**Keywords:** Helicopter emergency medical services, Interfacility transport, Critical care

## Introduction

A mechanically ventilated patient was transported by air ambulance from a community hospital intensive care unit (ICU) to a leading tertiary center. Door-to-door travel time was 42 min, which included 12 min airborne and 30 min of transfer time. With only 58 km between the referring and receiving hospital, a ground ambulance under similar conditions would have taken 46 min. While it is intuitive to believe that air is generally quicker and hence better than a ground transfer, was the decision appropriate? How should critical care physicians decide when to use a limited and expensive resource like helicopter emergency medical services (HEMS) for their patients?

To date, research on HEMS has focused almost exclusively on retrospective studies of “scene” transfers, where helicopter teams rescue patients from the out-of-hospital environment. However, interfacility transport (IFT), the flight between two hospital facilities, makes up over 80% of HEMS patient flights in Canada [1–3]. Given that the majority of HEMS IFT are for ICU patients and involve a door-to-door estimated driving time of less than 60 min [4], this editorial will discuss (1) whether HEMS IFT is indeed superior in terms of clinical outcomes, cost-effectiveness, and transport time as compared to ground emergency medical services (GEMS) and (2) the factors that ought to be considered when choosing the mode of transport for critically ill patients.

### Healthcare outcomes and utilization

The added benefit of IFT HEMS on healthcare outcomes is questionable. Since many HEMS systems are staffed with physicians [5, 6] (as opposed to basic life support (BLS) certified staff in GEMS), it is possible that HEMS patients may have improved outcomes from the care provided by the in-flight physician [7]. However, data from North America suggests that when both HEMS and GEMS patients receive care from advanced life support (ALS) certified providers, HEMS IFT does not decrease patient mortality, morbidity, or disability [7]. Furthermore, by controlling for injury severity and patient demographics, retrospective analyses have demonstrated that HEMS IFT does not reduce healthcare utilization, as measured by ICU and hospital length of stay [7, 8].

### Cost and cost-effectiveness

HEMS IFT is costly but is not necessarily cost-effective. In Canada, government funding for the Ontario air ambulance program, Ornge, totals over $135 million annually [9]. A recent systematic review attempting to investigate the cost of HEMS and GEMS suggests that HEMS costs more than $15,000 per trip and is up to seven times more expensive than GEMS, without imparting a patient benefit [10]. Furthermore, HEMS flights have a higher rate of fatal accidents than other forms of air travel, and HEMS aircrews have been considered the occupation with the highest rate of work-related fatal accidents [11]. Carbon emissions with HEMS use is greater as well as compared to other forms of road transport. Indeed, a single 4-h helicopter flight produces as much carbon emissions as a typical passenger car will emit in a year [12, 13]. Given that half of HEMS flights are classified as non-emergency [3], even minor reductions in medically inappropriate HEMS use could lead to meaningful savings.

### Predicted and actual transport time

While HEMS IFT may benefit critically ill patients traveling long distances, short-distance HEMS IFT is not necessarily faster than GEMS in regions where GEMS services are readily available [8]. Compared with GEMS, requests for HEMS are associated with longer response times at the referring hospital between the decision to transfer and departure [8, 14]. In facilities without on-site helipads, HEMS has also been shown to lead to longer total transport time [8]. Furthermore, Ornge HEMS IFT dispatch teams underestimate the actual time to definitive care in nearly 90% of cases [15]. On average, these flights arrive 71.5 min later than expected [15]. While local system considerations may limit the generalizability of such results, it does signify a need to re-evaluate the assumption that air ambulance is always faster.

## Conclusion

**Fig. 1:**
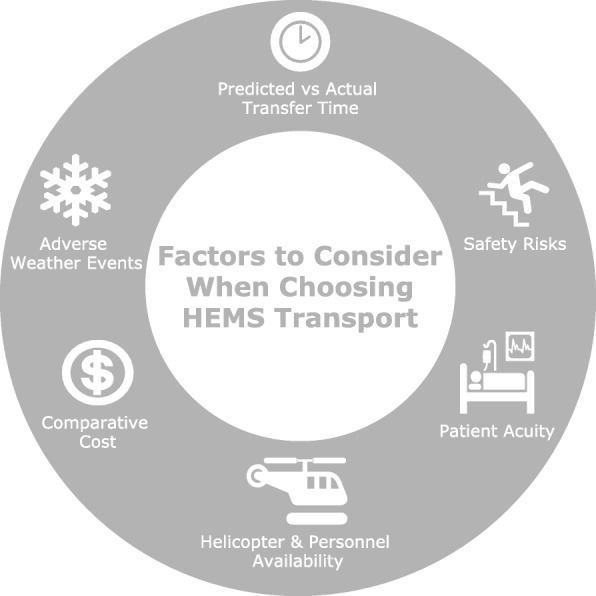
Factors to consider when choosing HEMS transport

Factors such as local weather conditions, the severity and urgency of patient illness, availability of trained personnel, and anticipated time for local HEMS dispatch as well as the likely duration of travel time should be considered before seeking HEMS (Fig. 1). For the most part, this local information is readily available when interfacility transfers are being discussed. Some degree of overtriage, flights wherein patients receive air ambulance transport despite not needing it, will always be inevitable, especially in scene transfers from an out-of-hospital environment in as yet unstable patients [1, 7]. During an IFT, however, physicians may have substantial diagnostic information and have stabilized their patients at the referring hospital, obviating the need for overtriage. It is, therefore, recommended that physicians at both referring and receiving hospitals communicate with each other, specifically about the mode of transfer that best suits the individual patient’s needs [11]. Policymakers and critical care physicians should be able to justify the use of this limited healthcare resource while advocating for the welfare of their patients.

## Data Availability

Not applicable

## Abbreviations

ICU: Intensive care unit
HEMS: Helicopter emergency medical services
IFT: Interfacility transport
GEM: Ground emergency medical services
ALS: Advanced life support
BLS: Basic life support
